# Phosphatidylserine Supplementation on Psychomotor Speed among Healthy Adults with Subjective Cognitive Declines

**DOI:** 10.1093/geroni/igaf122.3233

**Published:** 2025-12-31

**Authors:** Yin Liu, Yangfan Lu, Yunping Tang

**Affiliations:** Utah State University, Logan, Utah, United States; ECA Healthcare Inc., Shanghai, Shanghai, China (People’s Republic); Zhejiang Ocean University, Zhoushan, Zhejiang, China (People’s Republic)

## Abstract

About 12% of healthy adults in the U.S. have subjective cognitive decline (SCD). SCD coupled with fine motor impairment can increase risks of developing dementia. Supplementation with phosphatidylserine (PS), a fatty substance that improves blood lipid profiles, is associated with improved cognitive functioning in older adults and those with cognitive impairments. However, its benefits for fine motor functioning and SCD is not clear. We conducted a randomized clinical trial to evaluate the safety and potential efficacy of daily PS supplementation on psychomotor speed. The sample included 70 healthy Japanese who self-reported SCD at baseline (mean age = 50.23, sd = 8.68, range = 35-65). The 12-week trial used a stratified blocked randomized, double-blind, and parallel design. Participants received either placebo (n = 37), or DHAPS® product sourced from herring roe at 300 mg/day (n = 33). Computerized finger tapping test measured psychomotor speed. We conducted growth curve analysis to examine the main-effect on changes in psychomotor speed, and the moderating effect of time-varying omega-6/ omega-3 fatty acid ratio. We controlled time-invariant covariates on overall cognitive functioning and demographics measured at baseline. No adverse events were observed. Compared with that of the placebo group, the standardized motor speed (β = 37.73, se = 17.51, p = .03) was significantly faster in the DHAPS® group especially when the omega-6/omega-3 fatty acid ratio improved from the baseline. Regular daily consumption of DHAPS® at 300 mg/day as a nutritional supplement is safe and can benefit fine motor performance for healthy adults experiencing SCD.

